# Using a Social Justice and Health Framework to Assess European Climate Change Adaptation Strategies

**DOI:** 10.3390/ijerph111212389

**Published:** 2014-11-28

**Authors:** Melanie Boeckmann, Hajo Zeeb

**Affiliations:** 1 Department Prevention and Evaluation, Leibniz Institute for Prevention Research and Epidemiology-BIPS, Achterstr. 30, 28359 Bremen, Germany; E-Mail: zeeb@bips.uni-bremen.de; 2 Center for Social Policy Research, University of Bremen, Mary-Somerville-Str. 5, 28359 Bremen, Germany

**Keywords:** climate change, climate change adaptation, policy analysis, discourse analysis, social justice, public health, social determinants of health, Europe

## Abstract

Climate change puts pressure on existing health vulnerabilities through higher frequency of extreme weather events, changes in disease vector distribution or exacerbated air pollution. Climate change adaptation policies may hold potential to reduce societal inequities. We assessed the role of public health and social justice in European climate change adaptation using a three-fold approach: a document analysis, a critical discourse analysis of a subgroup of strategies, and a ranking of strategies against our social justice framework. The ranking approach favored planning that includes various adaptation types, social issues and infrastructure changes. Themes on values identified in the five subgroup documents showed that risks are perceived as contradictory, technology is viewed as savior, responsibilities need to be negotiated, and social justice is advocated by only a few countries. Of 21 strategy documents assessed overall, those from Austria, England and Sweden received the highest scores in the ranking. Our qualitative assessment showed that in European adaptation planning, progress could still be made through community involvement into adaptation decisions, consistent consideration of social and demographic determinants, and a stronger link between infrastructural adaptation and the health sector. Overall, a social justice framework can serve as an evaluation guideline for adaptation policy documents.

## 1. Introduction

### 1.1. Climate Change May Put Health at Risk

Climate change is a reality and may put human health at risk [1,2,3]. Projected climate change impacts include an increased frequency and intensity of heat waves and other extreme weather events, changes in disease vector and pollen distribution, or exacerbated air pollution [1,4,5]. Adverse health effects of climate change may include injuries and death following storms or floods, heat stroke and cardio-respiratory disease aggravation during extreme temperature events, an increased risk of infections and allergies, and higher skin cancer risks from increased UV exposure [1,5,6]. Governments in Europe thus face the need to prepare for these challenges, as has been supported by the Parma Declaration on Environment and Health in 2010 [7,8,9]. The term climate change adaptation describes measures undertaken to adjust to effects of climate, seeking to reduce harm and seize beneficial opportunities [10].

Adaptation has been described as a decision-making process that relies on effective governance [11]. In line with this definition, adaptation can be viewed as a task for all sectors and is not limited to environmental protection. Among previously identified climate change adaptation approaches, the following have been mentioned as promising for health protection: (1) monitoring and research, (2) consideration of demographic and social determinants, (3) community involvement, (4) early warning systems and emergency plans, (5) cross-sectoral efforts, and 6) infrastructural changes [12,13,14,15,16]. An integrated approach consisting of several of these measures has been described as more likely to protect health, regardless of the type of climate change impact [17,18].

### 1.2. Why a Social Justice Framework for Assessing European Climate Change Adaptation Strategies?

We assessed a sample of European climate change adaptation strategies with a framework informed by social justice concerns. Climate policy is subject to negotiations among priorities, perception and normative thinking [19]. Human health is but one of the concerns of climate-related policymaking. Yet, from a public health point of view, it seems odd that environmental protection should occur without explicit links to health, as the environment strongly influences health itself [20,21]. In addition, public health research has long been aware of the influence social environments have on human health [22].

Climate change affects human health not only directly but also indirectly through putting pressure on existing inequities and social determinants. Climatic changes have been linked to increased gender inequity [23,24], social disruption and forced cultural “re-inventions” [25]. Human health is doubly affected by climate change, once through direct effects on climate-related injuries and illness, and a second time through changes to socio-economic and cultural determinants of health [26,27]. The term “double exposure” [26] additionally implies that some population groups are twice burdened under a changing climate: first from existing inequities, and additionally through new risks imposed. Viewed through this lens, climate change adaptation becomes a tool with which not only the outcomes of climatic changes can be targeted, but which may assist in addressing social determinants of ill health. As such, climate adaptation may contribute to advancing social justice. Social justice matters because it lies at the heart of public health as a discipline [28], and can contribute to health protection [22].

Social and natural environment are difficult to separate: the complex interaction of factors contributing to health have led to the concept of “ecological public health” [29]. As social and cultural dimensions influence susceptibility to climate change related health effects [25,30,31,32], increased vulnerability to environmental risks as a consequence of climatic changes has been framed as an environmental health and justice issue [33,34,35,36].

An assessment of European climate change adaptation can profit from contrasting strategies against a social justice framework that is based on the understanding that:

- Unequally distributed social determinants of health create a situation of inequity among European populations [22];
- Climate change will exacerbate existing health risks [5];
- Adaptation aims to prevent negative impacts of climate change [37], and
- Adaptation measures can support health equity through targeting these social determinants of health.

As the official European climate change adaptation strategy also explicitly calls for the integration of social factors into adaptation activities [38], we consider a social justice framework an appropriate and useful concept to assess current European adaptation efforts.

## 2. Methods

The aim of our study was to assess the health protection potential of selected European climate change adaptation strategies from a critical policy appraisal perspective. Our approach was three-fold: a document analysis on recognized impacts and supported adaptation types of all 21 included strategy papers, a critical discourse analysis identifying themes on value statements of a subgroup of six strategies selected for their inclusion of social justice concerns during a keyword search selection process, and finally a ranking exercise of strategies within a health-focused social justice framework.

### 2.1. Document Analysis

A narrative review and document analysis of 21 European national adaptation strategies from 19 countries was conducted. We reviewed national adaptation strategies as specified on the European Environmental Agency (EEA) and European Commission (EC) joint website CLIMATE-ADAPT [39], complemented by an online search. The climate-adapt database is a useful source, because the European Environmental Agency aims at providing a comprehensive overview over all member states’ strategy approaches and has been included in the European Climate Change Adaptation Strategy [38]. To inquire about draft strategies published in English, we contacted countries with an adaptation strategy under development. The website CLIMATE-ADAPT lists 18 of 32 European countries with an adopted national adaptation strategy as of August 2014 [39]. These countries are Austria [40], Belgium [41], Denmark [42], Finland [43], France [44], Germany [45], Hungary [46], Ireland [47], Lithuania [48], the Netherlands [49], Portugal, Spain [50], Sweden [51], Switzerland [52], Turkey [53], and the United Kingdom [54,55,56]. Within the United Kingdom, Wales [54], Scotland [55] and England [56] provided individual strategies. The online search retrieved the Czech Republic’s national adaptation strategy [57] and a Norwegian report [58] on climate change adaptation. Slovakia only provided a background document via personal communication as the official strategy is still being developed. The Bank of Greece provided a general report on impacts and adaptation measures [59].

**Figure 1:**
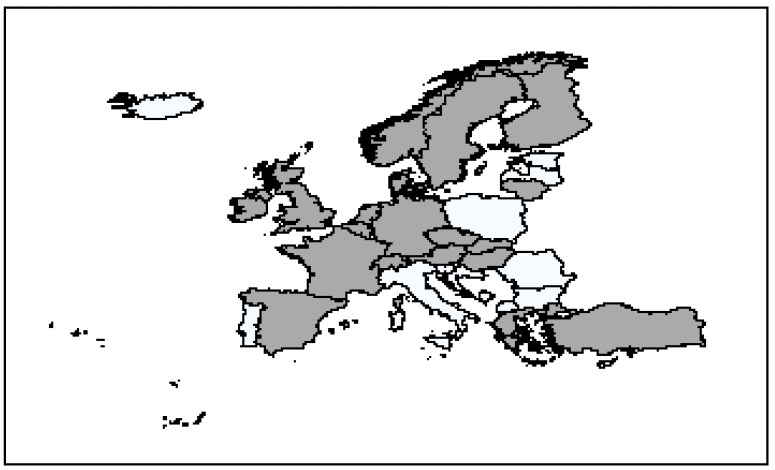
Map of European countries included in the study.

Notes: Documents from 19 countries (three from the UK) were included in this study. These countries are marked in dark color. © Eurogeographics [60] for the administrative boundaries.

Eight country strategy documents had to be excluded from analysis: Slovenia’s strategy document covers only the forest and agriculture sector. Estonia, Latvia and Italy are currently developing national strategies. Romania, Poland, Bulgaria and Portugal did not provide an English-language version of their strategies and had to be excluded from analysis. Figure 1 shows a map of the countries included in our assessment [60].

The search term human health was entered into each adaptation strategy document to assess whether or not adaptation takes place specifically in the health sector. With the aim of covering specific vulnerabilities related to age, migration, socio-economic disadvantages and gender [5,22], the following keywords were used in a second search within documents: social, socio* (* = allowing for all possible endings of the word), justice, fair, disadvantage, elder*, migra* (for migration, migrant, migrate), demograph*, divers* (except biodiversity), and gender. Sampling of strategy documents was driven by two considerations: First, we were interested in the framing of social determinants of climate change vulnerability in the official national document. Thus we excluded all documents that did not touch upon these issues. Second, we included only strategies in the subsample that contained the keywords fairness or justice.

We are aware that an *absence* carries meanings of its own. However, for the purposes of this research project, these absences of social justice consideration in strategies led to a lower ranking of the strategy and were not analyzed further. Six strategy documents from Austria, England, Finland, Greece, Sweden and Wales were included in our subgroup. The strategy texts were closely read and coded for themes stating values in MaxQDA software using a critical discourse analysis (CDA) approach influenced by van Dijk [61] and Fairclough and Fairclough [62].

### 2.2. Ranking of Country Strategies against the Social Justice Framework

Our ranking approach is based on adherence to the social justice framework with additional preference for infrastructural adaptation [63].

Bittner *et al*. [64] proposed a formula for ranking European heat warning systems. They assigned a value between 0 and 2 for stage of development of sub-parts of a heat warning system and added 25% to this partial score in cases where evaluation of the system took place. We propose an altered ranking method that takes into account:

- The high relevance of changes in social determinants of health under climate change [65], and
- The potential for successful health protection expected from structural adaptation [5].

Thus we argue for a higher weighting of those strategy documents that fare best when situated within the social justice framework. We assigned one point for each type of adaptation included in a national strategy. This is based on comprehensiveness of strategic approaches as our preferred concept for national adaptation efforts [66]. Subsequently, we added percentages to the partial scores as a weighting mechanism: 25% of the partial score for those documents explicitly addressing social justice and fairness (keyword search), 20% of the partial score to those documents addressing migration and demographic changes, two major drivers of structural health inequities [67,68], and 15% of the partial score to those including structural adaptation.

### 2.3. Critical Discourse Analysis Methodology

The goal of this discourse analysis was to analyze themes surrounding social issues and climate change adaptation that emerged from the texts. What is discourse analysis? It “involves the use of language data as evidence of social phenomena, theorizing language as communication, practice or selective constructions derived from accrued social meanings” ([69], p. 27). The textual data used for this analysis was selected from the pool of all 21 national adaptation strategies in this project as specified above. We first identified six strategy documents that discussed justice and social or cultural aspects of climate change and adaptation through a keyword search. Only documents containing the keywords justice or fairness and additionally migration or demographic changes were included in the subsample. In a second step, topics and value themes in these documents were analyzed following methods proposed by van Dijk [61] and Fairclough and Fairclough [62]. These methods are: close reading of the text, identification of topics and identification of themes related to values through an iterative process of coding, and memoing about these codes.

Fairclough and Fairclough [62] are interested in the power relations that drive the production of texts, using CDA to make conflicts and inequities visible [70]. Wodak and van Dijk stress the importance of context for analysis purposes [61,70]. Context exceeds the text itself and extends into socio-political realms [70]. This understanding makes van Dijk’s framework valuable for climate change studies: it has repeatedly been argued that the social context influences vulnerability, resilience and susceptibility to adverse effects of climatic changes [25,71]. Both approaches openly admit to having a political agenda, namely that of exposing mechanisms of social structures and identifying injustices [70].

Critical discourse theorists argue that knowledge can have different versions, some of which are accepted as truths and can be used to advance certain groups over others [69]. According to van Dijk [72], acceptance relies on access to dissemination of knowledge, for instance to media outlets. Official national documents may be perceived as prestigious and result in or prescribe specific actions, thus shaping the future of adaptation in each country. Consequently, exclusion or inclusion of social issues conveys an important message.

“Meanings are constituted through what is done” ([69], p. 10), therefore these documents show meanings attributed to health and social issues in national climate change adaptation strategies through what they suggest is done (as adaptation), *and* through the language and terms they are using.

Following Van Dijk’s approach [61], we searched for *topics* within texts to identify what a section of text represents, so that the principles behind the strategy documents could be elicited. In a second step, we identified *themes revolving around values*. Values play an important role in Fairclough and Fairclough’s practical reasoning framework [62]. Fairclough and Fairclough describe “practical reasoning [as] reasoning concerning what to *do* ” ([62], p. 35) (emphasis by the authors of this article). As this study aimed to assess climate change adaptation regarding its inclusion of and potential for health protection, the *actions* outlined in the strategy documents are of high interest. Processes of negotiation in climate change contexts have been discussed elsewhere [73,74] and are not part of this research project.

## 3. Results and Discussion

The document analysis revealed that all strategy documents include comments on health risks of climate change. Human health is a factor in the description of climate change impacts, adaptation options, or both.

### 3.1. Impacts of Climate Change on Health

**Figure 2:**
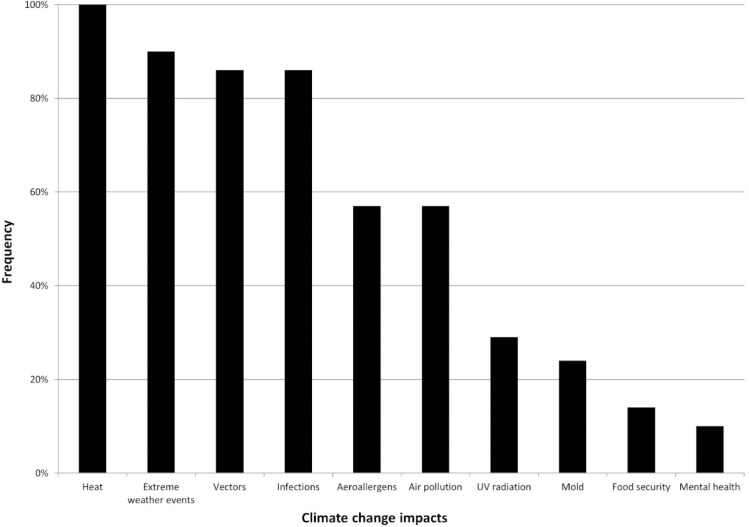
Impacts of climate change on health discussed in strategy documents ranked by frequency of inclusion in strategy documents (more than one impact was mentioned in each document). Heat and extreme weather events were mentioned separately in the texts, as were vectors and other infections. Infections refer to food- and water-borne infections.

Heat and extreme weather events play the largest role in European adaptation strategies, followed by infectious diseases (Figure 2). All 21 documents include heat, and 90% of documents discuss extreme weather events. Vector-, food- or water-borne infections are mentioned in 86% of the documents. Additional climate change impacts on health discussed are changes in aeroallergen distribution and exacerbation of air pollution (57% each), increase in UV radiation exposure (29%), mold development in houses (24%), food security (14%), and mental health issues (10%). Population displacement as results of climatic changes is only discussed in the Irish strategy.

### 3.2. Adaptation Measures in European Strategy Documents

We categorized adaptation into four major types, based on a typology proposed by Balbus *et al.* [75]:

- Data and surveillance
- Technological adaptation, including emergency plans and warning systems
- Behavioral adaptation and awareness raising
- Infrastructural adaptation

**Figure 3:**
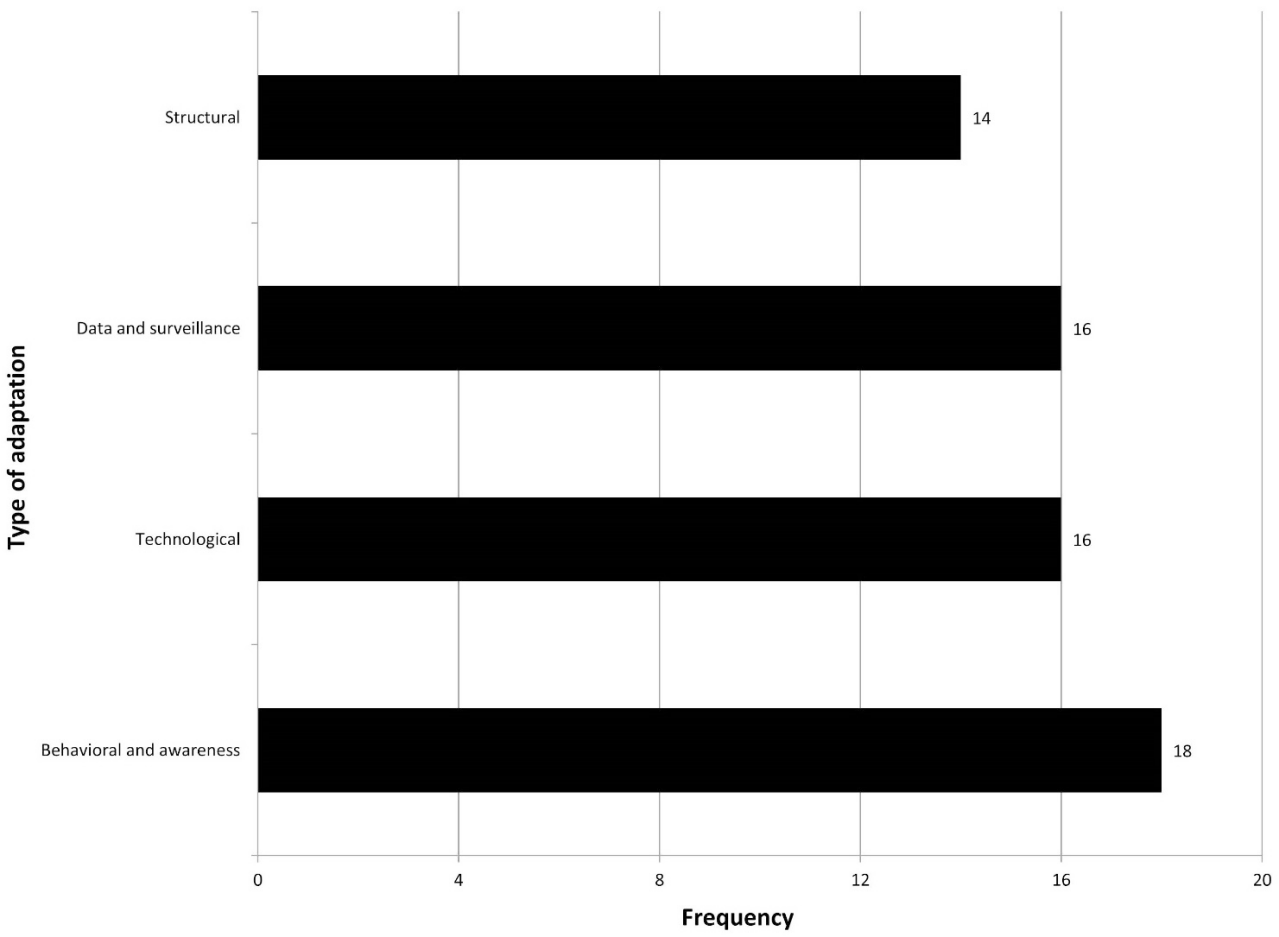
Types of adaptation proposed by strategy documents ranked by most frequent inclusion in strategy documents (more than one adaptation type was mentioned in each document).

When categorized according to type of adaptation, the most frequently cited adaptation type is awareness raising and education programs (18 documents), with technological adaptation and data/surveillance categories in 16 documents each (Figure 3). Infrastructural and engineering adaptation comes in last with 14 documents. Germany, Denmark, Hungary, and Turkey advocate vaccine development for emerging infectious diseases. Lithuania plans to strengthen health sector financing, and the Czech Republic stresses changes in European and national legislation as an additional strategy.

Adaptation to health impacts is proposed by all 21 strategy documents. Specificity and comprehensiveness of the proposed adaptation measures vary between the strategies. Slovakia, for instance, focusses on raising awareness among medical personnel and on implementation of a warning system. Turkey, on the other hand, includes all four types of adaptation in its planning. Of interest are innovative, structural adaptation measures proposed by strategies, such as building publicly accessible water fountains in Austria, adding air conditioning to hospitals in Sweden, or strengthening the National Health Service in England. Mainstreaming climate change adaptation into all policies is suggested by Austria.

Impacts such as food security, linked to climate impacts on global agriculture, mold development in private housing, UV radiation exposure, exacerbated air pollution, and mental health impairments after extreme events are mostly excluded from the adaptation descriptions. England suggests public UV monitoring. It is unclear whether this results from prioritization of other health impacts or from difficulties creating an adaptation measure for these risks. These risks in particular require trans-sectoral and societal approaches.

Not all country strategy documents view climate change as a threat: Norway and the Czech Republic position themselves as well-prepared for climate change. Despite a common awareness of climate change impacts and associated health risks, European adaptation programs differ in their assessment of potential consequences of these impacts.

The results of our document analysis show that adaptation measures for highly ranked risks such as extreme weather events, extreme temperatures and infectious diseases are persistently recommended in European national strategies. Beyond these common aims, however, varied levels of comprehensiveness occur between countries. These variations include both additional impacts recognized and adaptation measures proposed. A second variation can be found in the documents’ treatment of social issues, as discussed in the following paragraphs.

### 3.3. Ranking of Country Documents

Table 1 shows the ranking of the examined European national strategies against the social justice framework. The baseline partial score was calculated from number of adaptation types included; the more different types, the more comprehensive we judged the strategy to be. To this partial score we added weighting for social justice. Owing to this approach, Wales, with fewer adaptation types, did not rank in the upper two thirds despite its commitment to promoting fairness and equity.

Country strategies with the highest score are Austria, England and Sweden. These documents not only recommended all four types of adaptation measures but were additionally committed to promoting social justice, taking into account social determinants, and gained extra points for the inclusion of infrastructural adaptation. Six country documents rank in second place. In this ranking, we find Denmark, Lithuania and Scotland with a score below 2.5, followed by Ireland and the Netherlands.

### 3.4. Discourse Analysis of Subgroup Articles

Results of the CDA are represented as four value themes: (a) the cautionary principle in light of uncertainties, (b) responsibility, (c) technology as savior and (d) social justice and gender equity.

#### 3.4.1. The Cautionary Principle in Light of Uncertainties

Uncertainties are inherent to climate projections and lie at the heart of climate adaptation projects. Certain risks to human health have been described as generally applicable (such as extreme temperature, extreme weather events, and vector distribution changes, see also introduction of this article). Yet this universality of risks is handled differently among the examined countries. Heat to Finland [43] is both a risk and not a risk: a contradiction. Compare the following statements as an example:

*“Excess mortality is significantly higher in extremely cold temperatures than during periods of intense heat, and extremely cold temperatures are estimated to become less common,”*

([43], p. 157)

and:

*“[…][H]ealth impacts due to hot weather can be expected at lower temperatures in Finland compared to Central Europe”*.

([43], p. 157)

Here we observe an ambiguity in confronting an increase in extreme heat events.

**Table 1:** Ranking results.

Country	Type of Adaptation				Partial Score	Social Justice	Social Issues Migration and Demographic Change	Structural Adaptation	Total Score
	Data/Surveillance	Technological	Behavioral	Structural		Add 25%	Add 20%	Add 15%	
Austria	1	1	1	1	**4**	1	0.8	0.6	**6.4**
UK: England	1	1	1	1	**4**	1	0.8	0.6	**6.4**
Sweden	1	1	1	1	**4**	1	0.8	0.6	**6.4**
Belgium	1	1	1	1	**4**	0	0.8	0.6	**5.4**
Czech Republic	1	1	1	1	**4**	0	0.8	0.6	**5.4**
France	1	1	1	1	**4**	0	0.8	0.6	**5.4**
Germany	1	1	1	1	**4**	0	0.8	0.6	**5.4**
Norway	1	1	1	1	**4**	0	0.8	0.6	**5.4**
Turkey	1	1	1	1	**4**	0	0.8	0.6	**5.4**
Finland	1	1	0	1	**3**	0.75	0.6	0.45	**4.8**
Greece	1	1	0	1	**3**	0.75	0.6	0.45	**4.8**
Hungary	0	1	1	1	**3**	0	0.6	0.45	**4.05**
Switzerland	1	0	1	1	**3**	0	0.6	0.45	**4.05**
Slovakia	0	1	1	1	**3**	0	0	0.45	**3.45**
Spain	1	1	1	0	**3**	0	0	0	**3**
UK: Wales	0	1	1	0	**2**	0.5	0.4	0	**2.9**
Denmark	1	0	1	0	**2**	0	0.4	0	**2.4**
Lithuania	1	0	1	0	**2**	0	0.4	0	**2.4**
UK:Scotland	1	0	1	0	**2**	0	0.4	0	**2.4**
Ireland	0	1	1	0	**2**	0	0	0	**2**
Netherlands	0	0	0	0	**0**	0	0	0	**0**

A similar debate occurs regarding vector-borne diseases: On the one hand:

*“the climate has not been decisive for the occurrence of these communicable diseases or the emergence of a pathogen cycle,”*,

([43], pp. 158–159)

yet at the same time the Finish strategy suggest that ticks or bank voles may find more favorable conditions as a result of climate change ([43], p. 160).

These findings suggest that that careful consideration is of high value in Finland, with the goal of targeting the *right risks.* Overall, the contradictory nature of temperature-related risks does not deter Finland from acting: we would call this an adherence to the *precautionary principle*. This is illustrated in the following statement on reviews as part of an adaptation strategy:

*“[these are] the foundation for evaluating any no-regrets measures whose implementation would benefit the sector or target groups regardless of climate change”*.

([43], p. 11)

Benefits regardless of climate change as the ultimate justification for adaptation fits well into a precautionary framework.

This theme has also been picked up by Sweden [51]:

*“The warmer climate will affect health and lead to more deaths due to heat waves and increased spread of infection”*,

([51], p. 11)

later followed by:

“*Few cold snaps produce positive health effects”*.

([51], p. 430)

Acknowledging uncertainties leads to precautious activities in climate change adaptation. Policy acts in the face of scientific uncertainties, a theme that may be useful for social justice action among the lines of “better safe than sorry”.

#### 3.4.2. Who is Responsible for Adaptation?

A second theme prominent in the examined documents is global responsibility. European countries highlight contrasts between their positions and those of countries of the Global South, and formulate consequences of that positioning. For instance, Sweden [51] argues from a legal standpoint:

*“According to article 4.4 of the Climate Convention (UNFCCC), the industrialised countries (Annex I countries) should support the developing countries that are most vulnerable to climate change”*.

([51], p. 456)

Wales [54] takes this theme further and acknowledges that as an industrialized country in the Global North:

*“we are responsible for a much larger proportion of global emissions because of the goods and services we consume but which are made elsewhere”*.

([54], p. 15)

As a consequence, Wales proposes that:

*“Sharing experience and learning on this challenging agenda is vital and we are committed, through our Wales for Africa programme, to working with communities in other parts of the world in responding to climate change”*.

([54], p. 19)

England [56] similarly states that:

*“the government continues to support programmes helping the poorest and most vulnerable people in climate change ‘hot-spots’, as well as identifying and refining tools which are cost-effective and sustainable”*.

([56], p. 11)

Such commitment to “help” could also be interpreted as “othering” Africa and possibly additional countries in the Global South [76]. By creating a dichotomy of rich versus poor, technologically advanced versus helpless in the face of climate change, these European strategy documents may cement inequities rather than promote social justice. This interpretation is supported by a common perception that immigration is a result of climatic changes. The Austrian strategy states that:

*“studies on development mechanisms of migratory movements to Austria and Europe should be initiated to reduce or deal with possible migration”(translated from German by the authors)”*.

([40], p. 91)

Greece [59] *“has already received large numbers of immigrants, and these numbers will increase significantly in future as the flow of environmental refugees increases”*,

([59], p. 463)

and Sweden [51] concurs:

*“Sweden will also experience an increased number of cases of infectious diseases where the infection is contracted overseas due to increased global infection pressure”*.

([51], p. 443)

Immigration to European countries is discussed in the strategies and represents awareness about global migratory patterns.

The link between poverty and effects of climate change is generally acknowledged, leading to the above mentioned referral to Europe’s responsibility to mitigate climate change and support lower income countries. Incorporating environmental agreements into aid and development is Europe’s answer to these global inequities. Again, this approach has its shortcomings: within the UNFCCC negotiation processes, the least developed countries need to combine mitigation efforts and related expenses with national development goals [77]. Future research could examine this issue further and assess the implications between causing environmental harm first and subsequentlyoffering the “gift” of support to those being harmed in the process [78].

#### 3.4.3. Technology as Savior

Within the Welsh, English and Finnish strategy documents, technological adaptation is highlighted as the best solution, particularly for flood risks or in the shape of heat warning systems. As a value statement, inherent to technological solutions to climate change is solvency, *i.e*., being in a financially secure situation.

Finland [43] stresses that:

*“development of solvency is crucial to human health”,* and *“[t]he industrial-technical culture […] is capable of protecting human beings in various ways”*.

([43], p. 231)

England [56] agrees:

*“Development and economic progress will, in many cases, be the most effective way of helping countries to adapt, as well as helping to create stability”*,

([56], p. 11)

and proposes the development of new technology. Wales [54] is particularly ambitious in linking technology and climate change:

*“Ensuring that our approach to R&D, technology, innovation and skills help Wales gain maximum benefit from climate change related business and research”*.

([54], p. 6)

A technocratic solution also links back to the previous theme of responsibility and “othering”: while European countries are in the position to afford high-tech alternatives, the majority of countries might not be. A consequence could be a commitment to giving these technologies to the Global South, the implications of which have been discussed above. Beyond adaptation, technology plays an important role for mitigation with its promise of energy efficiency and “a new green deal [56].” In England and Wales, technology in adaptation is thus portrayed as promising economic opportunity, not only as a means to an end. The examined strategies value solvency, technological advancement and co-benefits of adaptation. The Greek document in particular points out financial gains as motivation for adaptation.

#### 3.4.4. Social Justice and Gender Equity

The theme of social justice is intricately linked to antidiscrimination, gender equity, fairness, and protecting cultural diversity. Austria and Wales specifically mention justice as a value and a goal.

Related to the issue of global responsibility, but equally applicable to the national context, Wales [54] acknowledges:

*“Climate change is a social justice issue. Globally, and here in Wales, we can expect its impacts to disproportionately affect those least able to manage them and who are, at the same time, least responsible for causing the problem”*.

([54], p. 16)

A clearly stated goal of the Welsh strategy is to:

*“[…] ensure that our policies to tackle climate change also promote social justice”*.

([54], p. 16)

Similarly, Austria [40] writes that policy development should weigh benefits and harms:

*“stratified by different population groups and gender” (translated from German by the authors)*.

([40], p. 44)

Regarding gender equity, the Austrian document proposes a commitment to enabling women to participate in adaptation processes:

*“It is important that even within climate change adaptation measures women receive equal opportunities to participate, create and decide in societal processes”(translated from German by the authors)* .

([40], p. 45)

However, neither strategy gives recommendations on specific actions to achieve social justice.

## 4. Discussion

### 4.1. Europe is Aware of Climate Change Health Impacts

In general, protecting human health is one goal of European climate change adaptation strategies. The impact assessments are in line with research results on projected impacts of climate change [1]. This is not surprising given that the strategy documents regularly refer to published research.

Our results confirm those of previous studies on human health as a vulnerable sector in national European adaptation strategies [66,79]. However, the link between health protection and climate change adaptation in other sectors is not always explicit. For instance, the Netherlands plans for flood risks, yet their strategy does not discuss health implications of structural adaptation.

Heat warning systems have recently been the subject of increased research activity [16,64], yet the national documents rarely described heat warning systems as specific projects. A possible explanation for this discrepancy may lie in national versus regional climate change adaptation approaches. A second reason might be the distribution of responsibilities between departments. And finally, heat warning systems are concrete outputs of adaptation projects, whereas national strategy documents serve the purpose of outlining a country’s overall approach to adaptation, specifying concrete actions in add-on documents. This has been done in Austria, Germany and France, for example, where action plans support the national strategies.

### 4.2. Social Determinants of Health Play a Role in European Climate Change Adaptation

Consideration of social and demographic determinants has been identified as an important aspect of climate adaptation [80]. In the examined adaptation strategies, social and socio-economic factors were considered in scenario design or impact analysis. Austria, Wales, England and Turkey acknowledged gender as a category that might contribute to (further) inequities. As we have seen in the discourse analysis, responsibility as a theme illustrates awareness of the interconnectedness of European countries with countries in the Global South. The role of social determinants of health is thus not limited to the local but extends beyond European borders. This may influence decision-making processes. It would be interesting to contrast these environmental strategies with official development aid documents and practices to see if values and goals are aligned between sectors. This might also shed light on whether solvency, a highly rated goal in England and Greece, extends to increasing solvency in the Global South. We also find it of interest that the precautionary principle plays such a large role in the discussion about climate change adaptation measures and human protection.

Six European adaptation strategies explicitly address climate change as a social justice issue, and 17 documents show awareness of migration and demographic changes as risk factors of climate change (Table 1). These results might be interpreted as promising; whether actual measures to reduce structural inequities will be taken remains to be seen. The large number of documents including proposals for structural adaptation might bear potential for health protection, as structural disease prevention programs have also been described as effective in environmental health [81]. Our novel ranking approach allowed us to combine assessments of justice and health protection potentials. However, any ranking has an inherent bias towards certain variables: the Czech Republic fared well in the ranking, yet the entire strategy only discusses health in one paragraph. Any ranking results should therefore not replace an in-depth analysis of policy documents before drawing conclusions.

### 4.3. Weaknesses of European Strategies Persist

Our results show a gap between current knowledge on good practice adaptation for health and specific actions in health policy, confirming previous research [8,66,82]. Not all good practice advice from research has been incorporated into European adaptation design. There seems to be potential for improvement in linking health and infrastructure or planning, especially for the climate risks of flooding and extreme heat as these are highly relevant for urban design and for the health sector. Very little consideration has been given to community involvement. Most national strategies examined here do not yet adequately design approaches for the inclusion of communities or adaptation target populations. Wales is a positive exception, focusing on local adaptation efforts. Overall, further research into appropriate forms of *participatory* adaptation in Europe seems warranted. Such research designs could draw from results of community-based adaptation projects in developing countries [71,83].

A large number of European strategies are not yet accompanied by action plans, indicating further potential for an improved adaptive response. Systematic considerations of uncertainties associated with climate change adaptation, from climate models to national socio-economic development, are still missing [66]. Uncertainties play a prominent role in climate change discourses: from estimating impacts [84] to evaluating policy [85], what we cannot know about the future shapes current responses to climate change. Living with these uncertainties might require policymakers to rethink standard approaches to evidence-based policy.

Within the policy documents, possible co-harms and co-benefits to health of proposed adaptation strategies are rarely explored. Previous research has suggested that any adaptation measure could lead to unwanted negative effects on health, such as changing walking behavior through urban planning adjustments [86]. Both negative and positive effects of strategic measures could also be modelled, as has been done for mitigation strategies [87].

### 4.4. What Is Next for European Adaptation Strategies?

Within the climate and health research community, new concepts have been proposed. “Planetary health” [88] stresses the links between global environments and human health. If we assume such a large scale interdependency, strategies that only propose isolated individual adaptation measures might not be sufficient. As stated before, evidence for the effectiveness of specific measures is still missing, despite hints at potential effects of adaptation [89,90]. Instead, viewing adaptation policy as a larger, transformational effort has been suggested [91]. Karen O’Brien distinguishes between unintended and deliberate transformation, and defines it as “*[…] physical and/or qualitative changes in form, structure or meaning-making that can also include a psycho-social process*” [91]. Applied to European adaptation strategies, transformation could mean trans-sectoral approaches, long-term visions, innovative solutions, and designing policies aimed at structural changes. We have seen that the examined strategies propose technological innovations, “green deals” and even “cultural re-inventions,” to borrow the term from Adger *et al.* [25]. Some ideas of transformational designs are already included in Europe’s strategies, yet the term itself is not used.

### 4.5. Limitations

This study has several limitations. Only national strategies and draft national documents were included in the analysis. Individual cities or regions might have different strategies in place. Local adaptation is likely more capable of taking regional differences and needs into account [92]. However, national legislation plays an important role in encouraging and enabling regional agencies to pursue adaptation [92]. Several European countries are currently developing strategies not yet included in this study. Climate change adaptation is a work in progress, and keeping track of updated documents is challenging.

Documents without an English or German translation had to be excluded. Six of nine strategy documents excluded are from Central and Eastern European countries. Their national adaptation strategies might stress different approaches for health protection not covered here.

Using a social justice framework of course implies our normative approach to climate change adaptation. Our ranking method and selection of themes mirrors this normative understanding and may not necessarily reflect the aims of the policymakers who developed the strategies.

Recent studies have reviewed European heat wave warning systems [16,64], finding a larger number of warning strategies in place than mentioned in official national strategy documents. The reason for this omission in official national documents is unclear.

As we selected the strategies for the discourse analysis based on their discussion of social determinants, we are necessarily biased to detect reasons for an inclusion of said determinants rather than reasons for exclusion. Strategies without social determinants do not state reasons for the omission. We argue that the absence in itself carries a local meaning: it could be interpreted as perceived lack of relevance and/or political will to engage with these social aspects of climate change adaptation. Additionally, our assessment was necessarily based on the data sources we used. Other documents from the selected countries may lead to different conclusions.

## 5. Conclusions

A social justice framework can serve as an evaluation guideline for adaptation policy documents. We were able to show that the links between social determinants of health and a potential exacerbation of inequities under climatic changes are partially acknowledged in European countries.

Drawing from previous research into evaluation of adaptation, we have developed a theory-driven method to portray most promising strategy documents for health protection through foregrounding social justice, social determinants of health, and structural adaptation measures. Our results can contribute to strengthening the focus on human health and reduction of injustices in adaptation efforts.

In this article we have repeatedly pointed out the necessity to ground any adaptation actions for health protection in a socially responsible framework.

Our results suggest that European adaptation strategies are aware of climate risks, including adverse effects on health. A large number of European countries have made strides in preparing for climate change and combine two or more adaptation types to address these risks. This study complements a recent WHO survey on health action and climate change in Europe [8] by adding a social justice dimension and qualitative assessment. In European countries, progress could still be made through community involvement into adaptation decisions, and consistent inclusion of social and demographic determinants. A stronger link between infrastructural adaptation and the health sector could be considered.
